# Medicinal ethnobotany in Huacareta (Chuquisaca, Bolivia)

**DOI:** 10.1186/1746-4269-8-29

**Published:** 2012-08-02

**Authors:** Rodrigo Quiroga, Lidia Meneses, Rainer W Bussmann

**Affiliations:** 1 Centro de Biodiversidad y Genética, Universidad Mayor de San Simón, Casilla 538, Cochabamba, Bolivia; 2 Museo de Historia Natural Alcides D’Orbigny, Casilla 4324, Cochabamba, Bolivia; 3 William L. Brown Center, Missouri Botanical Garden, P.O. Box 299, St. Louis, MO, 63166-0299, USA

## Abstract

**Background:**

The aim of this study was to document the types of diseases treated by the use of medicinal plants, their main applications and also to have a report of the major diseases treated at the Hospital of San Pablo de Huacareta (Chuquisaca Bolivia).

**Methods:**

We conducted semi-structured interviews on the use medicinal plants with 10 local informants, and categorized the kinds of diseases treated by traditional medicine. We obtained reports of cases treated at the Hospital of Huacareta in order to compare the use frequency of traditional medicine and allopathic medicine for the treatment of recurrent diseases in the area.

**Results:**

Our survey identified 258 traditional medicine uses, spanning a total of 13 diseases categories and including 91 native and exotic plant species and one unidentified sample plant type. Gastrointestinal disorders (55%) were most frequently treated with medicinal plants, followed by afflictions of the musculoskeletal system (25%) and dermatological disorders (24%). Hospital information indicates that the most common diseases are acute respiratory infections (47%) and acute diarrheal diseases (37%). The herbal remedies were mostly used in the form of teas and decoctions. The informants used mainly native plant species, although exotic species has been introduced to the pharmacopoeia.

**Conclusions:**

The treatment of gastrointestinal disorders is the primary objective of the medical ethnobotany of the inhabitants of Huacareta, while respiratory system diseases are mostly treated in the hospital. Looking at the data from the Hospital records we can infer that gastrointestinal disorders are among the most common diseases in the study area. For most respondents, traditional medicine is a reliable choice for the care of their illnesses. However, the preference of the population for either traditional medicine or allopathic medicine needs to be clarified in future comparative studies to obtain more convincing results. The results presented can be used as a base for subsequent work related to traditional medicine and its contribution to allopathic medicine in San Pablo de Huacareta.

**Resumen:**

**Introducción**

El objetivo del presente estudio fue documentar los tipos de enfermedades tratadas mediante el uso de plantas medicinales, sus aplicaciones principales y también tener un reporte de las enfermedades mayormente atendidas en el Hospital de San Pablo de Huacareta (Chuquisaca, Bolivia).

**Métodos:**

Se realizaron encuestas semiestructuradas a 10 informantes locales anotando los usos atribuidos a sus plantas medicinales, se agruparon las plantas por categorías de enfermedades tratadas en la medicina tradicional. Se obtuvieron reportes de casos tratados en el Hospital de Huacareta para poder relacionar el tratamiento de enfermedades recurrentes en la zona entre la medicina tradicional y la medicina occidental.

**Resultados:**

Se reportaron 91 especies nativas y exóticas, además de un espécimen indeterminado exótico que intervienen en un total de 258 aplicaciones medicinales, las cuales son empleadas en un total de 13 categorías de enfermedades. Los desórdenes gastrointestinales (55%) son mayormente tratados mediante plantas medicinales, seguidas de las afecciones al sistema esqueleto-muscular (25%) y enfermedades dermatológicas (24%). La información del Hospital indica que las enfermedades más frecuentes son Infecciones Respiratorias Agudas (47%) y Enfermedades Diarreicas Agudas (37%). Los remedios vegetales se emplean en forma de infusiones y cocciones principalmente. Se emplean mayormente plantas nativas, también se introdujo en la farmacopea médica el uso de plantas exóticas al lugar.

**Conclusiones:**

El tratamiento de trastornos gastrointestinales constituye el objetivo primordial de la etnobotánica médica de los habitantes de Huacareta, las enfermedades del sistema respiratorio, son mayormente tratadas en el Hospital. Observando los datos del libro de consultas del Hospital, se puede inferir que los desórdenes gastrointestinales están entre las enfermedades más frecuentes en el área estudiada. Para la mayoría de los entrevistados, la medicina tradicional es una opción confiable para la atención de sus enfermedades. Sin embargo, la preferencia de los habitantes entre la medicina tradicional y la medicina alopática podría ser clarificada a partir de futuros estudios comparativos que permitan obtener resultados más convincentes. Los resultados expuestos pueden ser usados como una base de datos para posteriores trabajos relacionados a la medicina tradicional y su contribución con la medicina alopática en San Pablo de Huacareta.

## Background

San Pablo de Huacareta is located in a transition zone of valleys between the Andes and the Bolivian Chaco. It contains an interesting blend of Chaco and Andean plant species. Likewise, the human population in the area is composed of people of mixed descent, Quechua and Guarani, whose culture is rooted in Chaco and Andean traditional knowledge, which is still maintained. [1,2]. In this sense, it is interesting to be able to evaluate the medicinal ethnobotany of a population who presents the characteristics previously exposed.

In Bolivia the impact of hospitals and health posts only became important for the general population after 1975, when the government increased their number by over 80%. Nevertheless, rural communities in Bolivia are still relying on traditional medicine to treat everyday illnesses [3]. The use of medicinal plants to treat a wide variety of diseases has been often noted [4-7]. According to the World Health Organization, up to 90% of the population in developing countries relies on traditional medicine and medicinal plants to meet primary health care need [8]. In spite of the permanent loss of cultural practices worldwide, and also in Bolivia, traditional medicine still is very much a part of daily life in the rural areas [3,9]. Therefore, research on traditional forms of medicine and the exploration of the possible use of medicinal plants in primary health care is required to understand the extent of use and effectiveness of these practices. There exists some interest at the international level to systematize the information on the use of medicinal plants for each region [1,10]. Previous research has shown that 80% of people in developing countries use traditional medicine to meet their primary healthcare needs, and about 85% of a traditional medicine involves the use of plant extracts [11].

No data on the medicinal plants used by local people in Huacareta existed prior to this study. Previous reports only indicated in general terms that the practice of traditional medicine was mainly rooted in cultural patterns and reflected through practices, beliefs, and customs [12].

Recent ethnomedicinal work in Bolivia highlighted the contribution and cultural appreciation of the traditional medicine by indigenous groups and mestizos. Most research has been conducted in Cochabamba [3,5,13-16]; Santa Cruz [17-19] and Tarija [7], and the ethnopharmacological uses of medicinal plants in rural indigenous or mestizo communities in Chuquisaca was virtually unknown [1].

It is important to keep in mind that the data obtained in this study come from interviews with mestizo informants who keep a daily practice of using traditional medicine. They obtained their knowledge from their parents and grandparents. The common ancestry establishes a single source Chaco ethnicity, because the cultural mix that exists in the area today this does not allow us to assess the original cultural origin of the traditional knowledge and local uses of medicinal plants correspond to a Hispanic, Quechua or Guarani tradition, given their geographical location of transition between the Andean valleys and Chaco. The ethnobotanical approach used here focuses on the study of the meanings plants acquire in a particular cultural framework. In a study of this nature, therefore, the medicinal uses of plants are contextualized within the various practices associated with curation as defined by local people. For this reason, we also present a preliminary outline of their ethnomedicinal practice to facilitate the understanding of the role assigned to the use of medicinal plants. The aim of this study was to document the types of diseases treated by the use of medicinal plants, their main applications and also to have a report of the major diseases treated at the Hospital of San Pablo de Huacareta.

### Research area climate and vegetation

**Figure 1:**
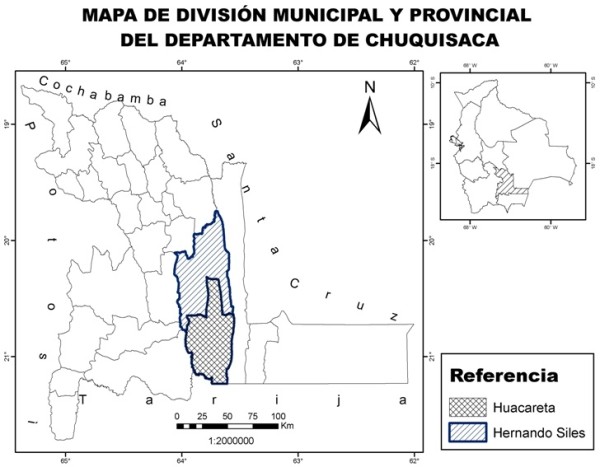
Location of the study area.

San Pablo de Huacareta (20° 21' 49.1" S, 63° 59' 59.3" W) is a town with roughly 2900 inhabitants of mixed mestizo (Spanish, Quechua, Guarani) origin and is located at the convergence of the Chuquisaca valley and the Bolivian Chaco (Santa Cruz, Chuquisaca, Tarija). Huacareta is located in the South of Hernando Siles province, in the Chuquisaca Department at an elevation of about 1090 m [1]. There are two access roads with daily transportation from Monteagudo and Entre Ríos, both medium-sized cities linked to larger cities, such as Santa Cruz, Tarija, and Yacuiba (Figure1).

The climate is xeric to sub-humid with rainfall of 900-1100 mm per year. The average maximum temperature is 25°C, the minimum average is 12.2°C. Extreme temperatures recorded in recent years were a minimum of -2°C and a maximum of 35°C. The land in the study area is mainly by used for agriculture and livestock. Important tree species are *Prosopis alba, Acacia aroma,* and *Celtis tala*[1,2].

### Ethnography

The early twentieth century saw the start of the migration of people of Quechua and mestizo origin to Azurduy, Zudañez, and Tomina provinces, attracted by the wealth offered by these regions. Data on the foundation of Canton Huacareta are imprecise, but there is reference to the first settlements between 1925 and 1945. As late as 1945, the population of San Pablo de Huacareta was primarily of Guarani origin, and the ownership of the territory was family based. However veterans of the Chaco War settled in the area and engaged in agricultural activities. These families of mixed or mestizo descent exercised patronage over the Guarani [20]. The Agrarian Reform of 1952 did not promote major changes in the structure of land tenure [1].Nowadays, the 2900 residents of Huacareta are mostly of mixed mestizo (Spanish, Quechua, and Guarani) descent, having come originally from the valleys of Chuquisaca and Bolivian Chaco (Santa Cruz, Chuquisaca, Tarija). These people are mostly Catholic and engage in agriculture and livestock production on the plains. Generally, farms in the area are dominated by maize (*Zea mays*) and to a lesser extent peppers (*Capsicum* sp.) and peanut (*Arachis hypogaea*), although in recent years there has been a tendency towards diversification, with the cultivation of beans (*Phaseolus vulgaris*) and even potato (*Solanum tuberosum*) [1,21]. These products are brought to major urban centers such as Santa Cruz, Sucre and Tarija. There is a tendency for the local population to migrate to the cities in search of new employment opportunities and economic prosperity [1].

### Health care

St. Paul's Hospital in Huacareta serves a small patient population and not everyone in the region use the hospital. Factors that hinder access to health services include the large distance between the center and the isolated communities, difficult access due to the flooding of the rivers in the rainy season and roads that are passable only at certain times of the year. The infrastructure and hospital equipment have deteriorated over the years and are no longer sufficient. However, patients covered by the SUMI (Seguro Universal Materno Infantil) have access to ambulance service, which allows transfers to more central health care facilities (e.g. the Hospital of Monteagudo). San Pablo runs a program of child immunization and supplements minerals and vitamins, as well as offering midwifery and birth control services. According to recent data, 36.4% of the population care for their health through traditional medicine, while the remaining population use the hospital [1].

## Methods

Ethnobotanical data were collected between May and November, 2010, on farms and hamlets near the town of San Pablo Huacareta. The areas surveyed were selected according to accessibility and willingness of residents to share their knowledge about the use of medicinal plants.Semi-structured interviews were conducted with 10 local informants (4 men and 6 women) after establishing prior informed consent. Their ages ranged between 55 and 70. Six informants were healers with medium experience practicing only occasionally at home, who obtained their knowledge from their parents and grandparents, who practiced as traditional healers. The remaining informants were constantly practicing traditional healers with broad experience, who retained extensive knowledge in the use and application of plant medicines. These healers attended to 30% of the population of San Pablo. One healer can serve up to ten patients a day, usually Monday through Friday and in special cases may also attend on the weekends. The local healers treat mainly the most common diseases, such as skin infections, gastrointestinal disorders such as diarrhea, stomachaches, colic and bladder problems, colds, and uro-genital diseases such as kidney stones, kidney infections and urinary infections [1].

Data about traditional medicinal plant use, including mode of preparation and application, diseases treated, recommended dosage and frequency and adequate treatment, were recorded [13]. The consensus criterion used to validate the data gathered was based in having at least two informants identify the same part of the same medicinal plant for the identical medicinal use [4].

We categorized the diseases reported in this investigation in accordance to Bárbara Frei and Susana Arrázola [5,22], whose study is based on human body parts affected by an illness (e.g. respiratory system, skin, gastrointestinal tract, circulatory system, etc.), that were treated through using medicinal plants. The characteristics of each disease category are explained in the results. In addition the Hospital San Pablo de Huacareta was visited to obtain data on the number of cases treated during the period of 2009-2010 (1669 cases) of the same diseases in order to compare the use of traditional medicine and Western medicine. Seventy-five patients at the hospital were randomly selected and interviewed about the factors influencing their preferences in the use of either the hospital or traditional medicine, after establishing oral prior informed consent.

Seventy five randomly selected patients were interviewed about their preference of either being treated at the hospital, or by using medicinal plants. Patients were asked ‘Do you prefer hospital attention or medicinal plants to treat your illness?” The respective interviews were conducted in the last week of fieldwork.

Vouchers of all plants were collected directly in the field with the assistance of two traditional healers. The plants were identified and specimens were deposited in the *Herbario Nacional Forestal Martín Cárdenas* in Cochabamba (BOLV) under the collection series RQ. The interviews were recorded in field notebooks and worksheets and later digitized.

## Results

A total of 258 medicinal uses were recorded for a total of 91 native and exotic plant species belonging to 40 families and one unidentified sample. These medicinal applications fell into a total of 13 disease categories. Each plant might be used to treat various diseases. For example, *Opuntia ficus-indica* was used to treat heat stroke, sunburn, yellow fever, renal problems and gastritis; *Acacia aroma* was used for wounds, muscle pain, cancer, liver problems, and gastritis. The same applies to the rest of the diseases and plants reported (Table 1, Table 2). Leaves, branches, roots, and bark were the most frequently used plant parts, with the resin and exudates used to a lesser extent.

**Table 1:** Percentage of diseases reported in the patient register of Hospital San Pablo de Huacareta

**Disease / Age-class**	**0-1**	**2-4**	**5-9**	**10-20**	**21-59**	**60+**	**Total cases**
Respiratory	33	32	7	10	12	6	47
Gastro-Intestinal	37	23	8	5	14	14	37
Dermatological	35	39	7	3	15	0	8
Musculo-skeletal	0	0	0	13	36	51	4
Uro-genital tract	20	0	14	16	12	38	3
Cardiovascular	0	0	0	7	0	93	1

**Figure 2:**
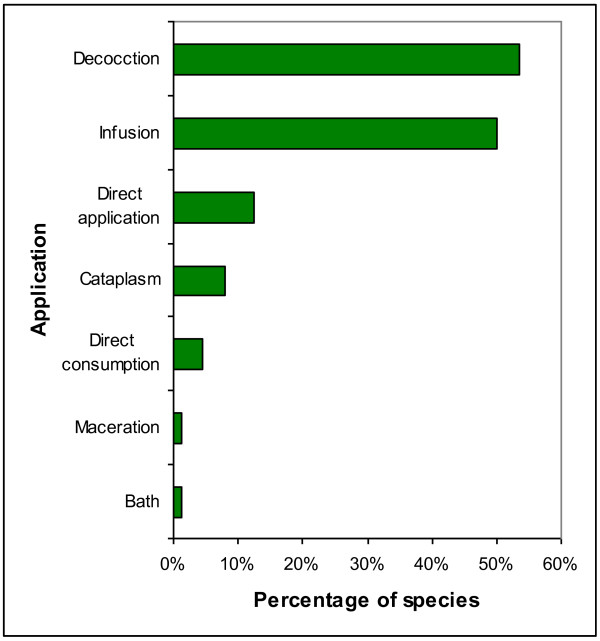
Mode of application.

Most remedies were prepared by simply boiling the ingredients (47 species, 53%) and were administered as tea (40 species, 50%). This was especially true for treating digestive disorders, liver and kidney; for which people rely on *Acacia aroma, Celtis tala, Mimosa debilis,* and *Equisetum giganteum*. For external afflictions, such as skin disorders, and musculo-skeletal pain, a poultice applied directly without any special preparation (11 species, 13%) was the application method of choice (Figure2). In some cases, plant parts like leaves and fruits were heated over a fire and then applied to the affected area (7 species, 8%), e.g., *Ricinus communis, Tipuana tipu, Pereskia sacharosa,* and *Sambucus peruviana*.

Native plants were mostly collected around the informants’ houses or in fields. Usually shrubs and herbs used in traditional medicine were found in agro-forestry plots.

### Major diseases treated with medicinal plants

#### Gastrointestinal disorders and liver disease

Gastrointestinal disorders (55% o the plants used), included diarrhea, dysentery, colic, spasms, gastritis, ulcers, nausea, vomiting and liver problems. These symptoms were often accompanied by pain, flatulence, loss of appetite, and fatigue. The most commonly used route of administration of remedies was by infusion. Often several species were combined in one preparation. Important species used to treat these disorders were *Acacia aroma*, *Psidium guineense*, *Celtis tala*, *Tecoma stans*, and *Verbena berteroi*. Leaves, roots, and branches were most frequently used for the treatment of the ailments mentioned.

#### Musculo-skeletal problems

Musculo-skeletal problems (25% of the plants used), included disorders and trauma associated with joints, muscles, or bones. The most widely used means of administration was the application of heated plant materials, often in a bath, which were derived from branches and leaves. Mixed leaves of *Schinus molle*, *S. longifolius,* and *Salix humboldtiana* were the most frequent remedy used.

#### Skin diseases

Skin diseases were treated with 24% of the plants used, and covered all diseases affecting the skin or mucous membranes, such as bacterial infections, eczema, dermatitis, acne, bleeding, burns, wounds, allergies, blisters, abscesses, and bites. They were often accompanied by symptoms described by the informants as pain, bleeding, itching, or swelling. Remedies were applied as a poultice or in a bath, in most cases. The most frequently used plant parts were leaves, roots, and branches of *Ricinus communis*, *Argemone mexicana*, and *Schinus longifolius*.

#### Uro-genital problems

The uro-genital disease complex (20% of the plants used) included ailments affecting both women and men, especially, reproductive system problems associated with childbirth and venereal diseases. Teas were the main forms of preparation for dealing with these ailments. The most widely used plant parts were leaves, roots, and branches of species such as *Cissus simsiana*, *Chamaesyce serpens*, *Plantago major*, and *Tecoma stans*. Kidney disorders treated were primarily kidney stones and urinary tract infections.

#### Respiratory problems

Ailments related to the respiratory tract were treated with 17% of all plants found, mostly in the form of teas. This included diseases of the throat and lungs, cough, colds, and flu. Autoimmune disorders like asthma were also classified as respiratory. The most widely used material were leaves, branches, and flowers from *Argemone mexicana*, *Eucalyptus globulus*, *Pluchea sagittalis*, and *Matricaria chamomilla.*

#### Fever and malaria

Fevers (including malaria) as well as conditions like heat stroke were treated with 16% of the medicinal plants encountered. These problems were addressed by cooking roots, branches, and leaves to prepare a tea for the patient or to apply the material as poultices. The most important species used were *Xanthium spinosum*, *Opuntia ficus-indica*, *Cereus validus*, and *Plantago major*.

#### Cardiovascular diseases

Cardiovascular diseases included disorders of the heart and circulatory system, as well as diseases described as “of the blood” by the local informants. Eight percent of the plants found were prepared as teas to treat cardiovascular problems. The leaves of *Citrus sinensis*, *Aloysia triphylla*, and *Melissa officinalis* were particularly important, and served often as blood purifier.

#### Central nervous system disorders

Central nervous system disorders (7%) were treated mostly by drinking tea. *Citrus delicious*, *Lactuca sativa*, and *Citrus aurantium* were most frequently used to relieve nervous stress.

#### Other diseases

Among the diseases treated to some extent with herbal medicine were diabetes, headache, earache, toothache, and viral diseases. In all cases, the remedies were administered as herbal teas. For the treatment of earache fruits of cotton (*Gossypium hirsutum*) were applied directly to the ear, after first heating them over a fire.

**Figure 3:**
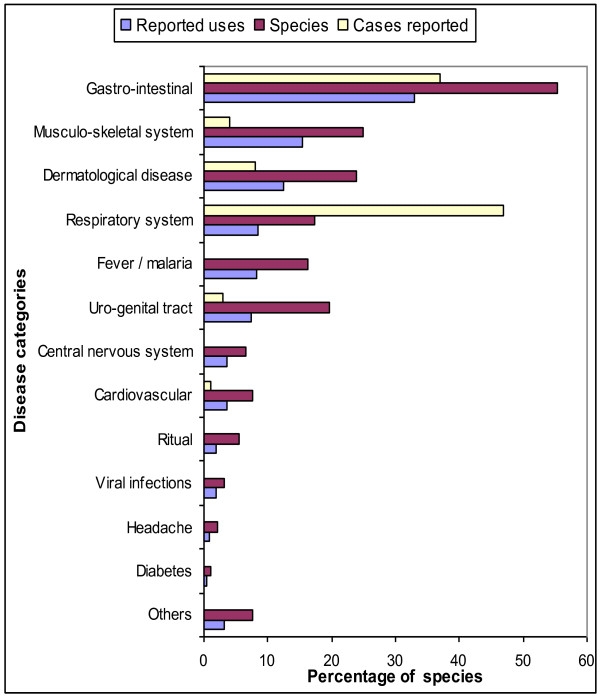
Percentage of species used and reported incidence of diseases.

Overall, we found that most plants are reportedly used for the treatment of gastrointestinal disorders (51 species, 55%), followed by plants used for musculoskeletal system disorders (23 species, 25%), skin diseases (22 species, 24%), and diseases of the genitourinary complex (18 species, 20%). Most applications subsequently dealt the treatment of gastrointestinal disorders (85 applications, 33%), disorders of the musculoskeletal system (40 uses, 16%), and dermatological diseases (32 uses, 12%) (Figure3).

Reports obtained from the Hospital San Pablo de Huacareta indicated that recurrent diseases in the area corresponding to the Acute Respiratory Infections-IRA-(47%), related to colds, coughs, laryngitis, pneumonia, diarrheal diseases-EDA-(37%), related to diarrhea, gastroenteritis, parasites, stomach pain, gastritis, skin diseases (8%) and cutaneous fungal infections, allergies, and diseases of the genitourinary complex (4%) and urinary tract infections, problems presented and post-partum (Figure3), the percentages herein are based on a total of 1669 cases reported throughout the year. Skeletto-muscular problems and skin diseases have a lower incidence (4% and 8% respectively) (Table1), these conditions can be cured by the use of medicinal plants, for example, a type of skin allergy is commonly called Tennis-court and is treated with resin *Morrenia odorata* (Table 2) and various diseases of the genitourinary complex are treated with medicinal plants (19 uses, 7%).

The numbers of uses attributed to herbal remedies to treat various ailments that afflict the population show the contemporary relevance of traditional medicine in Huacareta. Certain diseases like *Urifa* (dehydration in children) are usually treated with medicinal plants (e.g. *Schinus molle, Pereskia sacharosa, Mimosa debilis*), and treatment is often associated with a particular ritual. Likewise, some types of skin allergies are cured by a ritual and the use of medicinal plants, e.g. *Senecio* aff. *rudbeckiifolius*.

Although hospital records showed a higher incidence in the treatment of diseases of the respiratory tract and gastrointestinal disorders, we found that children ranging from 0-9 years had a higher incidence of cases of acute respiratory disorders (72% in total) and acute gastrointestinal problems (68% in total) within the total population attending the health center (Table1), the remainder corresponding to patients aged 10 and over 60 years of age. These data show the importance of the hospital in providing health care to children under the age of 10 years, who often suffer from diarrhea, intestinal parasites, and respiratory infections.

**Table 2:** Medicinal plant species encountered

**Voucher #**	**Family**	**Species**	**Vernacular name**	**Medicinal use**	**Disease Category**	**Part used**	**Preparation**	**Habit**	**Origin**
RQ147	Unidentified	unidentified	Cunfai	Digestion, gallbladder, Liver, diabetes, heart, kidneys	2, 3, 7	Leaves	Infusion	Herb	Introduced
RQ164	Adiantaceae	*Adiantum* sp.	Culandrillo	To avoid hair loss, post partum anti-inflammatory	3, 13	Entire plant	Infusion, decoction	Herb	Native
RQ157	Anacardiaceae	*Astronium urundeuva* (Allemão) Engl.	Sotillo	Bone pain, body pain, anesthetic, caries, fractures	8, 13	Bark, leaves	Decoction, cataplasm	Tree	Native
RQ142	Anacardiaceae	*Schinus longifolius* (Lindl.) Speng.	Chirimolle	Measles, smallpox, wounds, body pain	1, 8, 11	Leaves, branches	Cataplasm, bath	Tree	Native
RQ169	Anacardiaceae	*Schinus molle* L.	Molle	Body pain, cold, rheumatism, chagas, urifa (dehydration in children)	4, 6, 8	Leaves, branches, flowers	Decoction, Infusion	Tree	Introduced
RQ144	Apiaceae	*Foeniculum vulgare* Mill.	Hinojo	Digestion	2	Leaves, branches	Infusion	Herb	Introduced
RQ131	Apiaceae	*Petroselinum crispum* (Mill.) Fuss.	Perejil	Dehydration	5	Leaves, branches	Decoction	Herb	Native
RQ154	Apiaceae	*Hydrocotyle* sp.	Berro	Lungs, kidneys, liver, gastritis, spots in the face, regenerating	1, 2, 3, 4	Leaves	Eaten	Herb	Native
RQ205	Apiaceae	*Pimpinella anisum* L.	Anís	Stomach pain, des-inflammatory	2	Seed	Infusion	Herb	Introduced
RQ196	Apiaceae	*Apium graveolens* L.	Perejil	Stomach problems, cold	2, 4	Leaves, Roots	Infusion	Herb	Introduced
RQ210	Asclepiadaceae	*Morrenia odorata* (Hook. & Arn.) Lindl.	Supua	Cancha cancha (allergy), wounds	1	Resin, branches	Direct application, Infusion	Herb	Native
RQ171	Asteraceae	*Ambrosia tenuifolia* Spreng.	Artemisa	Body pain, to open pores, Malaria	1, 5, 8	Stem, leaves, entire plant	Decoction	Herb	Native
RQ177	Asteraceae	*Baccharis articulata* (Lam.) Pers.	Carqueja	Stomach problems, bitter taste in mouth, gallbladder, liver	2	Stem, leaves	Decoction	Herb	Native
RQ168	Asteraceae	*Baccharis dracunculifolia* DC.	Tola	Body pain	8	Leaves, branches	Decoction	Shrub	Native
RQ150	Asteraceae	*Bidens pseudocosmos* Sherff	Saiquilla	Liver	2	Flower, fruit	Decoction	Herb	Native
RQ134	Asteraceae	*Eupatorium* sp.		Cough	4	Flower	Infusion	Shrub	Native
RQ201	Asteraceae	*Lactuca sativa* L.	Lechuga	Relaxant	10	Leaves	Infusion	Herb	Introduced
RQ173	Asteraceae	*Matricaria chamomilla* L.	Manzanilla	Flu, colds, stomach pain	2, 4	Flower, branches	Infusion, decoction	Herb	Introduced
RQ167	Asteraceae	*Pluchea sagittalis* (Lam.) Cabrera.	Cuatro cantos	Liver, gallbladder, cold	2, 4	Stem, leaves, branches	Infusion, decoction	Herb	Native
RQ182	Asteraceae	*Senecio* aff. *rudbeckiifolius* Meyen & Walp.	Maicha	Allergies, ritual	1, 9	Branches	Direct application	Herb	Native
RQ137	Asteraceae	*Xanthium spinosum* L.	Amor seco	Molar pain, heat, aft, hangover, fever, stomach pain, muscle pain, sterility, measles	2, 3, 5, 8, 11, 13	Roots, leaves, branches, entire plant	Infusion, decoction	Herb	Native
RQ213	Bignoniaceae	*Tabebuia impetiginosa* (Mart ex DC)	Lapacho rosado	Liver	2	Bark	Decoction	Tree	Native
RQ212	Bignoniaceae	*Tabebuia lapacho* (K. um. Sandw)	Lapacho amarillo	Kidneys	3	Bark	Decoction	Tree	Native
RQ121	Bignoniaceae	*Tecoma stans* (L.) Juss. ex Kunth.	Guaranguay	Liver, stomach pain, kidney, gallbladder, hangover	2	Leaves	Infusion	Tree	Native
RQ140	Brassicaceae	*Coronopus didymus* (L.) Sm.	Chanca piedra	Tumors, pustules, spots on the face, wounds, liver, kidneys	1, 2, 3, 8	Leaves, branches	Infusion	Herb	Native
RQ138	Cactaceae	*Cereus validus* Haw.	Ulala	Sunstroke	5	Mucilage	Direct application	Cactus	Native
RQ139	Cactaceae	*Opuntia ficus-indica* (L.) Mill.	Tuna	Sunstroke, sunburn, yellow fever, renal problems, gastritis	2, 3, 5	Exudates	Cataplasm, bath, infusion	Cactus	Native
RQ214	Cactaceae	*Pereskia sacharosa* Griseb.	Sacharosa	Pang (muscle pain), sore muscles, urifa (dehydration in children)	8, 9	Spines, leaves	Infusion	Cactus	Native
RQ189	Caprifoliaceae	*Sambucus peruviana* Kunth	Sauco	Heat, muscle pain, wounds, tumors, intestinal and stomach inflammation	1, 2, 8, 13	Leaves, fruit	Eaten, decoction, Infusion	Tree	Native
RQ191	Caricaceae	*Carica papaya* L.	Papaya	Anti-parasitic, stomach inflammation, purgative, gallbladder	2	Seeds	Eaten, Direct application	Tree	Introduced
RQ126	Chenopodiaceae	*Chenopodium ambrosioides* L.	Paico	Stomach pain, swollen stomach, cold, acidity, diarrhea	2, 4	Leaves, branches, flower, Stem	Infusion	Herb	Native
RQ125	Equisetaceae	*Equisetum giganteum* L.	Cola de caballo	Diarrhea, stomach heat, liver, kidneys (des-inflammatory)	2, 3	Stem, entire plant	Decoction, drink	Herb	Native
RQ178	Euphorbiaceae	*Chamaesyce serpens* (Kunth) Small	Chanca piedra	Kidneys	3	Entire plant	Infusion	Herb	Native
RQ130	Euphorbiaceae	*Jatropha curcas* L.	Piñón	Wounds	1	Resin	Direct application	Herb	Native
RQ183	Euphorbiaceae	*Manihot esculenta* Crantz	Yuca	Diarrhea	2	Roots	Infusion	Herb	Introduced
RQ149	Euphorbiaceae	*Ricinus communis* L.	Tártago	Carachas (scar skin), pustules on the head, swellings	1	Leaves, fruit, oil	Decoction, direct application	Shrub	Native
RQ117	Fabaceae	*Acacia aroma* Gillies ex Hook & Arn.	Sirao	Wounds, muscle pain, liver, gastritis	1, 2, 8	Leaves, Bark	Decoction, direct application	Tree	Native
RQ159	Fabaceae	*Anadenanthera colubrina* (Vell. Conc.) Benth	Vilca	Diarrhea, rheumatism, body pain	2, 8	Bark, resin	Decoction	Tree	Native
RQ133	Fabaceae	*Mimosa debilis* Humb. & Bonpl. ex Willd.	Celosita	Pancreas, heart, purifying, nerves, urifa (dehydration in children)	2, 6, 9, 10	Branches, roots	Decoction	Shrub	Native
RQ156	Fabaceae	*Myroxylon peruiferum* L.f.	Quina	Bone pain	8	Bark, resin	Decoction	Tree	Native
RQ136	Fabaceae	*Prosopis alba* Griseb.	Algarrobo	Cloudy eyes, asthma, twisted bones	1, 4, 8	Leaves, Bark	Decoction, infusion, cataplasm, direct application	Tree	Native
RQ211	Fabaceae	*Tipuana tipu* (Benth.) Kuntze	Tipa	Gastritis, wounds	1, 2	Bark	Infusion	Tree	Native
RQ187	Iridaceae	*Sisyrinchium chilense* Hook.	Linasa	Cold, heat, sunstroke	4, 5	Fruit	Infusion	Herb	Introduced
RQ145	Lamiaceae	*Melissa officinalis* L.	Toronjil	Heart, cold, nerves, stomach, dysentery, headache	2, 4, 6, 12	Leaves, branches	Infusion, decoction	Herb	Introduced
RQ195	Lamiaceae	*Mentha x piperita* L.	Hierba buena	Stomach gas, stomach pain, digestive	2	Leaves	Infusion	Herb	Introduced
RQ197	Lamiaceae	*Ocimum basilicum* L.	Albahaca	Digestive, cold	2, 4	Leaves	Infusion	Herb	Introduced
RQ209	Lamiaceae	*Origanum vulgare* L.	Orégano	Stomach pain, menstrual pain	2, 3	Branches	Infusion	Herb	Introduced
RQ204	Lamiaceae	*Pulegium* sp.	Poleo	Stomach pain	2	Leaves	Infusion	Herb	Native
RQ186	Lauraceae	*Cinnamomum zeylanicum* Blume	Canela	Cold, diarrhea	2, 4	Bark	Infusion	Herb	Introduced
RQ190	Liliaceae	*Aloe vera* L.	Sábila	Wounds, spots, pimples in the face, gastritis, fever, sunstroke, muscle pain,	1, 2, 5, 8	Exudates	Direct application, infusion	Herb	Introduced
RQ184	Lythraceae	*Heimia salicifolia* Link	Paraguay	To wash the head	1	Leaves	Infusion	Herb	Native
RQ141	Malvaceae	*Gossypium hirsutum* L.	Algodón	Ear pain, deafness	13	Fruit	Direct application after heating in fire	Tree	Introduced
RQ198	Malvaceae	*Malva parviflora* L.	Malva	Liver, gastritis, stomach problems, renal inflammation, diuretic	2, 3	Leaves	Infusion	Herb	Introduced
RQ163	Malvaceae	*Sida rodrigoi* Monteiro	Huacachi	Stomach swelling	2	Roots	Decoction	Shrub	Native
RQ208	Myrtaceae	*Blepharocalyx salicifolius* (Kunth) O. Berg	Arrayán	Bone pain, body pain, postpartum	3, 8	Branches, bark	Decoction	Shrub	Native
RQ192	Myrtaceae	*Eucalyptus globulus* Labill.	Eucalipto	Asthma, sinusitis	4	Leaves	Decoction, steam	Tree	Introduced
RQ146	Myrtaceae	*Myrcianthes callicoma* McVaugh	Sahuinto	Digestion	2	Leaves, bark	Infusion, decoction	Tree	Native
RQ202	Myrtaceae	*Psidium guajava* L.	Guayaba	Diarrhea	2	Leaves	Infusion	Tree	Introduced
RQ123	Myrtaceae	*Psidium guineense* Sw.	Guayabilla	Diarrhea	2	Roots, leaves	Decoction	Tree	Introduced
RQ165	Nyctaginaceae	*Pisonia ambigua* Heimerl	Coso coso	Intestinal parasites of children	2	Flower	Decoction	Herb	Native
RQ148	Papaveraeceae	*Argemone mexicana* L.	Cardosanto	Cough, wounds, stomach anti-inflammatory	1, 2, 4	Flower	Infusion	Herb	Native
RQ175	Passifloraceae	*Passiflora cincinnata* Mast.	Murucuya	Allergies	1	Entire plant	Decoction	Herb	Native
RQ152	Phytolacaceae	*Petiveria alliacea* L.	Ánamo	Cold, fever, swellings, rheumatism, bone and muscle pains	3, 5, 8	Roots, leaves	Infusion, decoction	Herb	Native
RQ153	Piperaceae	*Piper acutifolium* Ruiz & Pav.	Matico	Cold, Cough, wound disinfectant, bone pains, rheumatism	1, 4, 8	Leaves	Infusion, decoction, cataplasm	Herb	Native
RQ120	Plantaginaceae	*Plantago major* L.	Llantén	Heat, sunstroke, liver, gallbladder	2, 5	Roots	Decoction	Herb	Native
RQ188	Poaceae	*Triticum aestivum* L.	Trigo	Smallpox and measles	11	Seeds	Decoction	Herb	Introduced
RQ199	Poaceae	*Zea mays* L.	Choclo	Diuretic, cold	3, 4	Styles	Infusion	Herb	Introduced
RQ174	Polygonaceae	*Coccoloba tiliaceae* Lindau	Banduro	Wounds	1	Leaves	Decoction	Shrub	Native
RQ172	Rhamnaceae	*Condalia weberbaueri* Perkins	Yana yana	Sunstroke, heatstroke	5	Leaves, branches	Decoction	Shrub	Native
RQ179	Rosaceae	*Prunus persica* L. (Batsch)	Durazno	Sunstroke, headache	5, 12	Leaves	Direct application	Tree	Introduced
RQ151	Rosaceae	*Rubus boliviensis* Focke	Zarzamora	Rheumatism, swellings, dysentery, diarrhea	2, 8	Roots, leaves	Infusion, decoction	Shrub	Native
RQ162	Rutaceae	*Citrus x aurantifolia* (Christm.) Swingle	Limoncillo	To quench thirst	5	Fruit	Eaten	Tree	Native
RQ207	Rutaceae	*Citrus aurantium* L.	Naranja agria	Nerves, to wash the head	1, 10	Leaves	Infusion, decoction	Tree	Native
RQ193	Rutaceae	*Citrus deliciosa* Ten.	Mandarina criolla	Nerves	10	Leaves	Infusion	Tree	Native
RQ203	Rutaceae	*Citrus limon* (L.) Burm.f.	Limón	Gallbladder, colic	2	Fruit	Eaten	Tree	Introduced
RQ194	Rutaceae	*Citrus maxima* (Burm.) Merr.	Pomelo	Gastritis, stomach burning, acidity, stomach pain	2	Leaves, fruit	Infusion, Eaten	Tree	Introduced
RQ185	Rutaceae	*Citrus sinensis* (L.) Osbeck	Naranja	Stomach anti-inflammatory, colds, heart	2, 4, 6	Leaves, flower	Infusion	Tree	Introduced
RQ127	Rutaceae	*Ruta graveolens* L.	Ruda	Neck pain, ear pain	8, 13	Leaves, branches, entire plant	Infusion, cataplasm, direct application	Herb	Native
RQ170	Salicaceae	*Salix humboldtiana* Willd.	Sauce	Body pain, cold	4, 8	Leaves, branches	Decoction	Tree	Native
RQ135	Smilacaceae	*Smilax* sp.	Candelillo	Bladder infection, kidneys, stomach inflammation, wounds	1, 2, 3	Roots	Decoction	Shrub	Native
RQ129	Solanaceae	*Brugmansia* sp.	Floripondio	Allergies, abortion, pain, wounds	1, 3	Leaves, flower	Decoction, cataplasm	Shrub	Native
RQ128	Solanaceae	*Cestrum parqui* Benth.	Yerba santa	Stomach infection, dysentery, colic, urifa (dehydration in children)	2, 9	Roots, leaves, branches	Decoction	Herb	Native
RQ181	Solanaceae	*Nicotiana glauca* Graham	Carallanta	Hemorrhoids, muscle swelling	1, 8	Leaves	Decoction	Herb	Native
RQ206	Solanaceae	*Solanum tuberosum* L.	Papa	Gastritis	2	Roots	Infusion	Herb	Introduced
RQ132	Tiliaceae	*Triumfetta semitriloba* Bojer	Cabeza de negro	Heat, sunstroke, purifying	5, 6	Roots	Decoction	Herb	Native
RQ180	Ulmaceae	*Celtis pallida* Torr.	Tala	Diarrhea	2	Bark, leaves	Infusion	Shrub	Native
RQ124	Ulmaceae	*Celtis tala* Gill.	Tala	Diarrhea	2	Bark, leaves	Infusion	Shrub	Native
RQ122	Urticaceae	*Urera baccifera* (L.) Gaudich.	Itapallo	Liver, rheumatism, allergy, paralysis	1, 2, 8, 10	Roots, leaves, entire plant	Direct application, poultice	Herb	Native
RQ143	Verbenaceae	*Aloysia triphylla* Royle	Cedrón	Heart, cold, nerves	4, 6, 10	Leaves	Infusion, decoction	Tree	Introduced
RQ116	Verbenaceae	*Verbena berteroi* (Meisn.) Schauer	Verbena	Swellings, blows, fever, intestinal problems	2, 5, 8	Leaves, entire plant	Decoction	Herb	Native
RQ176	Verbenaceae	*Vebena hispida* Ruiz & Pav.	Verbena	Liver	2	Branches	Decoction	Herb	Native
RQ118	Vitaceae	*Cissus simsiana* Schult. & Schult. f.	Zarzaparrilla	Liver, stomach anti-inflammatory, kidney, purifying	2, 3	Roots	Decoction	Herb	Native

Legend: 1: Dermatological disease; 2: Gastro-intestinal; 3: Uro-genital tract; 4: Respiratory system; 5: Fever/malaria; 6: Cardiovascular; 7: Diabetes; 8: Músculo-skeletal system; 9: Ritual; 10: Central nervous system; 11: Viral infections; 12: Headache; 13: Others.

In informal conversations 67 out of 75 informants mentioned a preference for the use of medicinal plants instead of going to the hospital. This could mainly be linked to the distrust that people have in doctors. Another important aspect was the limited financial resources available for the purchase of pharmaceuticals. Often people consult doctors at the hospital, then turn to traditional healers for treatment with medicinal plants, because this cure has a lower cost. However, the preference for traditional medicine over allopathic medicine needs to be further investigated with a larger number of interviews.

### Native and exotic plants

Within the research area 68% of all plants used (63 species) were native (i.e., they occurred naturally in the study area) and were applied in 69% of all reported remedies (179 uses). The most important plant species were *Xanthium spinosum* (9 applications), *Coronopus didymus*, *Petiveria alliacaea*, *Piper* sp., *Hydrocotyle* sp., and *Verbena berteroi* (6 applications each), Te*coma stans, Urera baccifera, Chenopodium ambrosioides, Brugmansia* sp., *Xanthium spinosum* and *Rubus boliviensis* (5 applications each), *Acacia aroma, Plantago major, Equisetum giganteum, Pluchea sagittalis, Baccharis articulata*, and *Ruta graveolens* (4 applications each).

The remaining 32% (29 species) were exotic plants, (i.e. introduced species), like *Eucalyptus globulus*, often planted as part of reforestation efforts, and were used in 31% of all applications (78). The most prominent species were *Carica papaya* and *Citrus maxima* (4 applications), *Matricaria chamomilla, Prunus persica*, and *Mentha* sp. (3 applications) and *Gossypium hirsutum, Schinus molle, Sisyrinchium chilense, Triticum aestivum, Eucalyptus globulus, Pimpinella anisum, Origanum vulgare*, and *Zea mays* (2 applications each). Some informants purchased the exotic species (e.g., wheat, oregano, cinnamon) in the local market. The rest were cultivated in home gardens or in fields.

## Discussion

Digestive system disorders are very common, especially in rural areas, in particular in the Andes, the Amazon and the Chaco. Numerous papers on medical ethnobotany explain the use of medicinal plants for the treatment of these conditions in the valleys and the Chaco region of Bolivia [3,5,7,17-19]. Unsurprisingly, the informants used most of the medicinal plants reported to treat such digestive system diseases, particularly diarrhea, gastritis, and liver problems. It is important to note that most drinking water in the area comes from natural sources such as streams, and there is no drinking water treatment. The water is however regarded as safe, as it passes through a process of natural filtration and is supposedly taken from clean sources [1]. This does not, however, guarantee non-contamination, and reports of diarrheal diseases that cause infant mortality in Huacareta are frequent [1,23].

It is remarkable to find that the highest percentage of plants (55%) and applications (85 applications, 33%) intended to treat gastrointestinal disorders. In addition, natural remedies were used for treating diarrhea, stomach pain and liver. Similar plant use was observed in other rural areas of the Bolivian Chaco, indicating the importance of traditional medicine in the treatment of gastrointestinal disorders [7,17,19]. It needs to be emphasized, however, that better water treatment would be the most important step towards the eradication of major health problems such as diarrhea and dysentery in rural areas of developing countries such as Bolivia, Argentina, or Paraguay [4].

In addition to gastro-intestinal problems, the healers in the area most frequently treated respiratory infections, wound infections, as well as allergies [1]. The consumption of fluids (teas and decoctions), as well as use of poultices and the direct application of plant material producing a cooling effect on the patient's body in order to treat body aches, skin diseases and fever has commonly been reported [24,25].

The data obtained in the hospital indicate that intestinal and respiratory diseases are the most commonly reported conditions. Acute respiratory infections are usually treated by doctors at the hospital, while the traditional use of medicinal plants is restricted to the treatment of common colds and cough. Patients who used allopathic medicine dod often also consult traditional healers, as an alternative in particular to reduce costs of treatment. Similar trends have been observed among communities in Bolivian’s inter-Andean valleys and the Bolivian Amazon [2,5,14].

The main reasons for the informants (67 of 75) to prefer traditional medicine, are low income, lack of confidence in western doctors at the hospital, and also the long tradition to use plant based medicine [1]. Studies in Cochabamba [15,16] conform to the present study, indicating that customs, the effectiveness of traditional healers, and dissatisfaction with the hospital doctors are reasons to continue the use traditional medicine.

Some studies in the area indicate that 36.4% meet their health through traditional medicine and the remainders in formal centers [1], the main causes are the inaccessibility or availability of financial resources, lack of transportation, distance, and a higher confidence in traditional healers. The Hospital of Huacareta has now begun a project where doctors and traditional practitioners cooperate in the treatment of patients [1]. Previous research in other regions indicated already that such an approach is feasible [13,21,22]. The Hospital has 48 general practitioners, 9 midwives and works now with at least 5 traditional healers [1]. Patients come for Western treatments and also consult traditional healers, especially for a healing alternative to the use of medicinal plants and to reduce costs. A similar approach was observed in the Cochabamba Valley (Apillapampa ) [3]. When the disease is chronic, patients often opt for the purchase of drugs, in particular if they receive social security benefits.

We found that plant species collected by the population of Huacareta from the forest areas and fields surrounding the house are mostly native species (68%), while only 32% are exotics. The latter are grown in gardens or, like *Eucalyptus globulus*, for reforestation [26], and patients often buy these species in the local market or from other cities and towns. Similar results were found in traditional creole medicine in the northwestern Argentine Chaco [4], where 79% of the used species were native and 21% exotic. Many of the species used were emplyed to treat digestive system disorders. A more detailed study [6] showed that 78% of medicinally used species were wild collected plants, 8.5% cultivated, and 8.5% purchased in the market, and the remaining 5% were either grown in gardens or purchased.

## Conclusions

In this study we found that Huacareta inhabitants use ethnomedicine mostly for the treatment of gastrointestinal disorders. Their pharmacopoeia contains a wide range of herbal remedies for these afflictions, while diseases of the respiratory system are only treated with a small number of medicinal plants. For these diseases patients prefer to go to the hospital. Looking at the data from the Hospital records, we found that gastrointestinal disorders are among the most common diseases in the study area. In addition we found that cases of diarrhoea and parasitoses in children under 5 years are mostly treated with allopathic medicine. Most respondents indicated that traditional medicine is the best option in the care of their illnesses. However, the preference of the population for either traditional medicine or allopathic medicine should be better clarified through future comparative studies.Herbal remedies are mainly used in liquid applications, such as teas and consist mostly of native plants that grow in the fields and along paths in the area. Some exotics were introduced into the pharmacopoeia and are now are cultivated in home gardens and sold in the market.

The results presented in this paper can be used as a base for future work related to the traditional use of medicinal plants and their contribution to allopathic medicine in San Pablo de Huacareta.

## Competing interests

The authors declare no competing interests.

## Authors' contributions

RQ designed the research study, conducted fieldwork, analyzed the data and wrote the draft manuscript. LM assisted with field work, provided comments and suggestions on the draft manuscript. RB revised and translated the manuscript. All authors read and approved the final manuscript.

## References

[B1] Ortíz F Saracho F Honorable Alcaldía Municipal de Huacareta 2007 Sucre, Bolivia: Plan de Desarrollo Municipal

[B2] Navarro G Maldonado M Geografía ecológica de Bolivia 2002 Vegetación y ambientes acuáticos Cochabamba, Bolivia: Centro de Ecología Simón I. Patiño-Departamento de Difusión

[B3] Vandebroek I Thomas E Sanca S Van Damme P Van Puyvelde L De Kimpe N Comparison of health conditions treated with traditional and biomedical healthcare in a Quechua community in rural Bolivia J Ethnobiol Ethnomed 2008 4 1 10.1186/1746-4269-4-1 18194568 PMC2265262

[B4] Scarpa GF Plantas empleadas contra trastornos digestivos en la medicina tradicional criolla del Chaco noroccidental Dominguezia 2002 18 1 36 50

[B5] Arrázola S Atahuachi M Saravia E López A Diversidad florística medicinal y potencial etnofarmacológico de las plantas de los Valles Secos de Cochabamba Revista Boliviana de Ecologia y Conservacion Ambiental 2002 12 53 85

[B6] Scarpa GF Medicinal plants used by the Criollos of Northwestern Argentine Chaco J Ethnopharmacol 2004 91 115 135 10.1016/j.jep.2003.12.003 15036479

[B7] Quiroga R Arrázola S Tórrez E Diversidad florística medicinal y usos locales en el pueblo Weenhayek de la Provincia Gran Chaco, Tarija-Bolivia Revista Boliviana de Ecologia y Conservacion Ambiental 2009 25 25 39

[B8] WHO (World Health Organization) Traditional medicine –growing needs and potential WHO Policy Perspectives on Medicines 2002 2 1 6

[B9] Arenas P Fortunato R, Bacigalupo N Proceedings del VI Congreso Latinoamericano de Botánica Expectativas de los sectores sociales respecto a la etnobotánica 1998 Mar del Plata, Argentina: Missouri Botanical Garden Press 207 208

[B10] Varela BG Fernández T Taira C Cerda Zolezzi P Ricco RA Caldas López E Alvarez E Gurni AA Hajos S Wagner ML El “muérdago criollo” Ligaria cuneifolia (R & P) Tiegh. -Loranthaceae-. Desde el uso popular hacia el estudio de los efectos farmacológicos Dominguezia 2001 17 31 50

[B11] Farnsworth N Soejarto D Okerele O, Heywood V, Synge H The Conservation of Medicinal Plants Global Importance of Medicinal Plants 1988 WHO, WWF. UICN 25 51

[B12] Saracho R Ortiz F López T Honorable Alcaldía Municipal de Huacareta 2007 Sucre, Bolivia: Plan de Desarrollo Municipal. Estrategias adicionales

[B13] Hinojosa I Uzquiano E Floweres YJ Los Yuracaré: su conocimiento, experiencia y la utilización de recursos vegetales en el río Chapare 2001 FONAMA. EIA, La Paz, Bolivia

[B14] Vandebroek I Van Damme P Van Puyvelde L Arrázola S De Kimpe N A comparison of tradicional healers’ medicinal plant knowledge in the Bolivian Andes and Amazon Soc Sci Med 2004 59 837 849 10.1016/j.socscimed.2003.11.030 15177839

[B15] Ureña C Diversidad, clasificación y uso de plantas medicinales en la comunidad de Apillapampa de la provincia Capinota del departamento de Cochabamba 2001 Universidad Mayor de San Simón, Cochabamba: Tesis de Maestría en Ciencias Ambientales

[B16] Thomas E Quantitative Ethnobotanical Research on Knowledge and Use of Plants for Livelihood among Quechua, Yuracaré and Trinitario Communities in the Andes and Amazon Regions of Bolivia PhD thesis 2008 Faculty of Bioscience Engineering, Ghent University: Belgium 516

[B17] Gallo V Plantas Medicinales de los Guaranies Aporte al Conocimiento de la Etnobotánica Isoceña Guaraní en relación a su flowera Medicinal 1996 1 1 263

[B18] Montaño G Estudio etnobotánico y comparativo de tres comunidades Guaraní del Alto y Bajo Izozog, Provincia Cordillera 1997 Universidad Autónoma Gabriel René Moreno: Santa Cruz-Bolivia. Tesis para optar el título de Licenciatrura en Ciencias Biológicas

[B19] Bourdy G de Michel LR C Roca-Coulthard A Pharmacopoeia in a shamanistic society: the Izoceño-Guaraní (Bolivian Chaco) J Ethnopharmacol 2004 91 189 208 10.1016/j.jep.2003.09.013 15120439

[B20] Querejazu R Historia de la Guerra del Chaco 1998 La Paz, Bolivia: Editorial La Juventud

[B21] Arteaga M Perez B Moira L Bolivia. Atlas de Municipios CID 2000 La Paz, Bolivia: Instituto Nacional de Estadística

[B22] Frei B Baltisberger M Altamirano R Ariza R Lopea R Medical Ethnobotany of the Zapotecs of the Isthmus-Sierra (Oxaca-Mexico: Documentation and assessment of indigenous uses J Ethnopharmacol 1998 62 149 165 10.1016/S0378-8741(98)00051-8 9741887

[B23] Remez L Children under age five account for half of all deaths in Bolivia, with diarrhea the main cause Int Fam Plan Perspect 1990 16 115 116 10.2307/2133311

[B24] Pestalozii H Flora Ilustrada Altoandina 1998 Cochabamba: Herbario Nacional de Bolivia-Herbario Forestal de Bolvia-Universitat Bern

[B25] Sturzenegger O Enfermedad mental en un mundo arcaico. Documenta Laboris Año 5, n° 72. Progbranch de investigaciones sobre Epidemiología Psiquiátrica 1985 Buenos Aires: CONICET

[B26] Serrano M Terán J Identificación de especies vegetales en Chuquisaca. Teoría práctica y resultados 2000 INTERCOOPERATION, Fundación Ceibo. Sucre, Bolivia

